# A Comprehensive Clinical Decision Support System for the Early Diagnosis of Axial Spondyloarthritis: Multi-Sequence MRI, Clinical Risk Integration, and Explainable Segmentation

**DOI:** 10.3390/diagnostics16071037

**Published:** 2026-03-30

**Authors:** Fatih Tarakci, Ilker Ali Ozkan, Musa Dogan, Halil Ozer, Dilek Tezcan, Sema Yilmaz

**Affiliations:** 1 Department of Computer Engineering, Graduate School of Natural and Applied Sciences, Selcuk University, Konya 42130, Turkey; fatihtarakcii@gmail.com; 2 Department of Computer Engineering, Faculty of Technology, Selcuk University, Konya 42130, Turkey; musa.dogan@selcuk.edu.tr; 3 Department of Radiology, Faculty of Medicine, Selcuk University, Konya 42130, Turkey; drhalilozer@gmail.com; 4 Division of Rheumatology, Department of Internal Medicine, Gulhane Training and Research Hospital, University of Health Sciences, Ankara 06010, Turkey; dr_dilekturan@hotmail.com; 5 Division of Rheumatology, Department of Internal Medicine, Faculty of Medicine, Selcuk University, Konya 42130, Turkey; drsemayilmaz@hotmail.com

**Keywords:** axial spondyloarthritis, early diagnosis, deep learning, machine learning, transfer learning, segmentation, magnetic resonance imaging, decision support system

## Abstract

**Background/Objectives:** This study aims to develop a comprehensive Clinical Decision Support System (CDSS) that integrates multi-sequence sacroiliac joint (SIJ) MRIs with rheumatological, clinical, and laboratory findings into the decision-making process for the early diagnosis of axial spondyloarthritis (axSpA), incorporating segmentation-supported explainability. **Methods:** Multi-sequence SIJ MRI data (T1-WI, T2-WI, STIR, and PD-WI) were analysed from 367 participants (*n* = 193 axSpA; *n* = 174 non-axSpA controls). Sequence-based classification was performed using VGG16, ResNet50, DenseNet121, and InceptionV3 models; additionally, a lightweight and parameter-efficient SacroNet architecture was developed. Slice-level probability scores were converted to patient-level scores using the Dynamic Top-K Averaging method. Image-based scores were combined with a logistic regression-based clinical risk score using weighted linear integration (0.60 image/0.40 clinical) and a conservative threshold (τ = 0.70). Grad-CAM was applied for visual interpretability. Furthermore, to support the diagnostic outcomes with precise spatial data, active inflammation in STIR and T2-WI sequences was segmented. For this purpose, the MDC-UNet model was employed and compared with baseline U-Net derivatives. **Results:** Sequence-specific analysis showed VGG16 performing best on T1-WI (AUC = 0.920; Accuracy = 0.878) and DenseNet121 on STIR (AUC = 0.793; Accuracy = 0.771). The SacroNet architecture provided competitive classification performance at the patient level despite its low number of parameters (~110 K). Furthermore, MDC-UNet successfully segmented active inflammation, yielding Dice scores of 0.752 (HD95: 19.25) for STIR and 0.682 (HD95: 26.21) for T2-WI. **Conclusions:** The findings demonstrate that patient-level decision integration based on multi-sequence MRI, when used in conjunction with clinical risk scoring and segmentation-assisted interpretability, can provide a feasible and interpretable DSS framework for the early diagnosis of axSpA.

## 1. Introduction

**Figure 1:**
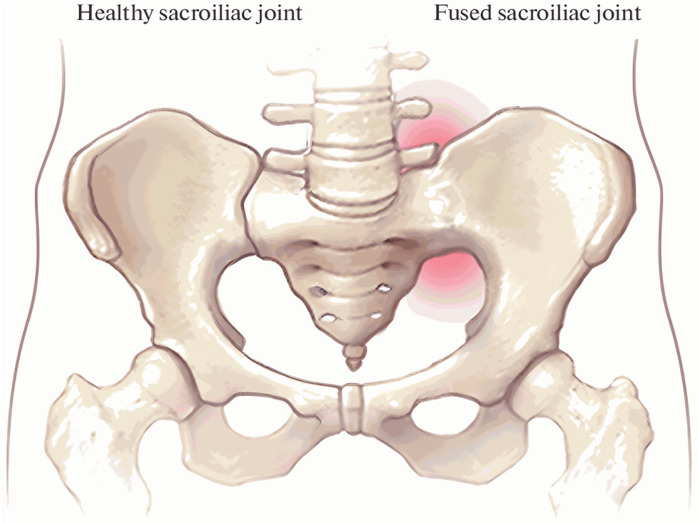
Comparison of a healthy SIJ with an inflamed and fused joint [6].

Axial spondyloarthritis (axSpA) is a chronic and progressive inflammatory rheumatic disease characterised by inflammatory back pain in the axial skeleton, affecting the spine and sacroiliac joints (SIJ) [1]. axSpA encompasses a clinical spectrum ranging from conventional radiography showing structural/radiographic evidence of sacroiliitis (radiographic axSpA) (r-axSpA), and non-radiographic axSpA (nr-axSpA), which lacks definitive radiographic evidence of sacroiliitis on conventional radiography but is clinically and/or MRI-based evidence suggestive of axSpA [2]. The disease typically presents before the age of 45, with the onset age generally between 20 and 30 years [3]. The global prevalence of axSpA varies depending on the classification criteria used, study design, and geographical/phenotypic characteristics; however, studies generally report a prevalence of approximately 0.36–0.70%, indicating significant heterogeneity between regions [4]. The core clinical signs and symptoms of axSpA include inflammatory pain, stiffness, and impaired mobility in the axial region and peripheral joints [5]. Chronic inflammation in the sacroiliac joints and spine causes back pain and stiffness. Over time, this process can lead to pathological new bone formation and structural damage in some patients, ultimately resulting in fusion of the sacroiliac joints and spine [3] (Figure 1).

The diagnosis of axSpA is made by evaluating clinical findings, laboratory data and imaging methods together. The main reason for this is that axSpA presents a heterogeneous clinical picture and no single finding obtained from the patient’s history, physical examination, laboratory tests or imaging studies is sufficiently sensitive or specific on its own to establish the diagnosis [7]. In this context, laboratory tests are considered as supportive tools in the diagnostic process, and there is no specific laboratory test for axSpA. Although the HLA-B27 test plays an important role in diagnostic evaluation, HLA-B27 positivity alone does not confirm the diagnosis of axSpA; similarly, HLA-B27 negativity does not rule out the possibility of axSpA [3]. HLA-B27 is a strong genetic predisposition marker, particularly for radiographic axSpA (r-axSpA), but results must be interpreted within a clinical context due to significant variations in population frequency across geographical regions and subgroups [8]. On the other hand, serum C-reactive protein (CRP) levels may be elevated in a significant proportion of patients with axSpA, but elevated CRP is neither sufficiently sensitive nor specific for diagnosis [9]. This situation increases the importance of imaging methods in diagnostic evaluation. Imaging methods play a critical role in the diagnostic evaluation of axSpA. Although conventional radiography and computed tomography (CT) can show structural changes, they are limited in detecting early inflammatory lesions [10]. Magnetic resonance imaging (MRI) is considered one of the most sensitive methods for the early diagnosis of axSpA, as it can show active inflammation in the early stages [11]. However, as the diagnostic contribution of MRI depends on the interpreter, the standardised and consistent evaluation of images is critical. The interpretation of MRIs requires advanced expertise [12]. Although MRI findings have been defined by the ASAS/OMERACT working group, limited agreement among readers leads to subjectivity and variability in the diagnostic assessment of axSpA [13,14].

The diagnosis of axSpA may be delayed compared to many other rheumatic diseases. A systematic review reports that diagnostic delay can extend up to 8 years [15]. However, it has been reported that treatment strategies applied to patients with early and accurate diagnosis reduce symptoms and slow disease progression [16]. In this context, there is a growing need for automated methods that increase diagnostic accuracy and consistency, reduce observer dependence, and can be integrated into clinical workflows.

In recent years, artificial intelligence (AI) approaches based on machine learning (ML) and deep learning (DL) have effectively supported diagnostic decision-making processes by enabling the quantitative analysis of complex patterns in medical images [17,18,19]. These developments have increased interest in AI-based clinical decision support systems (CDSS), particularly for diseases where early diagnosis is critical.

This study aims to develop a comprehensive CDSS based on the integration of multi-sequence MRI data with rheumatological, clinical, and laboratory findings, investigating its role in the early diagnosis of axSpA. The main contributions of this study are summarised as follows:

- We evaluate and compare the patient-level classification performance of DL models trained separately on T1-weighted imaging (T1-WI), T2-weighted imaging (T2-WI), STIR (Short Tau Inversion Recovery) and proton density-weighted imaging (PD-WI) MRI sequences.
- We evaluate the diagnostic contribution at the patient level of our proposed SacroNet model, developed specifically for axSpA, by comparing it with common transfer learning (TL)-based models. The novelty of the proposed model lies in offering a CNN-based, low-parameter/lightweight architecture trained from scratch as an alternative to common TL-based models. This design aims to reduce overfitting under limited data conditions, thereby learning patterns specific to axSpA in a more generalisable manner.
- By combining probability scores at the MRI slice level using a dynamic Top-K averaging strategy, we generate a single final diagnostic probability at the patient level. This enables a comprehensive evaluation of all slices for each patient, providing a patient-level assessment that is closer to the interpretation approach used in clinical practice.
- We combine image-based DL outputs with an ML-based clinical risk score to form the final diagnostic decision.
- In patients classified as axSpA, we enhance clinical interpretability by visualising potential active regions associated with inflammation using our proposed MDC-UNet-based DL model for segmentation purposes. The novelty of the proposed model, compared to the standard U-Net and common variants, lies in its hybrid integration of multi-scale dilated context extraction and attention mechanisms on the encoder side to adapt to the variable scale and distribution of SIJ payments, while preserving the encoder–decoder skeleton of the classic U-Net. Thus, it aims to increase the sensitivity of small lesions and make the boundary placement more accurate/stable.
- We anticipate that our proposed approach will provide a comprehensive methodological framework aimed at reducing specialist dependency in axSpA diagnosis and standardising the diagnostic process.

The contributions presented in this study are discussed within the framework of the current literature, which highlights the increasing importance of ML and DL-based approaches in axSpA diagnosis. Relevant studies are systematically presented in the following section.

### Related Works

AI-based approaches, especially when used with MRI, offer significant potential for the early diagnosis of axSpA [20,21]. Numerous studies using ML and DL methods for the diagnosis and assessment of AS have been reported in the literature. These studies are generally examined in two main groups: approaches focusing solely on classification and approaches addressing both classification and segmentation processes.

Classification-based studies:

The modality of classification-based studies aimed at automatically determining the presence or disease activity of axSpA consists of clinical data and imaging-based methods. In a significant portion of image-based studies, the SIJ is localized using manually defined regions of interest (ROIs), and ML and DL methods are widely used. A detailed comparison of these studies is presented in Supplementary Table S1 in the Electronic Supplementary Material (ESM).

Studies addressing classification and segmentation together:

In approaches that address classification and segmentation together, the presence of disease is automatically classified, while the SIJ or active inflammatory regions are visualised with segmentation outputs. A review of the relevant studies shows that U-Net and derivative methods are predominantly used in these approaches, and STIR and T1-WI MRI sequences are particularly preferred. A detailed comparison of these approaches is provided in Supplementary Table S2.

When these two tables are considered together, it is evident that a significant portion of the studies in the literature focus on a single MRI sequence and, in most cases, slice-level assessment; in contrast, end-to-end decision support approaches that integrate image-based outputs with clinical/laboratory variables using multi-sequence fusion at the patient level remain relatively limited. This highlights the need for integrated and explainable CDSS frameworks that better reflect clinical decision-making practice in the diagnosis of early-stage axSpA.

In addition to these studies in the literature, the findings of two review studies published in the field were also evaluated. One review reported that end-to-end DSSs, which integrate image-based DL, segmentation, and clinical risk factors at the patient level, remain limited [22]. The other review noted that studies in the literature are largely based on a single MRI sequence and limited to slice-level classification models [23].

The current literature highlights the potential of AI-based classification and segmentation approaches in axSpA diagnosis. This study integrates DL-based classification outputs evaluating multi-sequence SIJ MRI data at the patient level with an ML-based clinical risk score at the decision level; it also supports clinical interpretability by visualising active inflammation regions using U-Net-based segmentation outputs. Thus, an integrated and explainable AI-supported CDSS framework compatible with the clinical workflow for early-stage axSpA diagnosis is presented.

## 2. Methods

### 2.1. Ethics Statement

This retrospective study was conducted with approval from the Ethics Committee of Selçuk University Faculty of Medicine. All data were anonymised prior to analysis. The study was conducted in accordance with the ethical principles outlined in the Declaration of Helsinki [24].

### 2.2. Study Participants and Datasets

**Figure 2:**
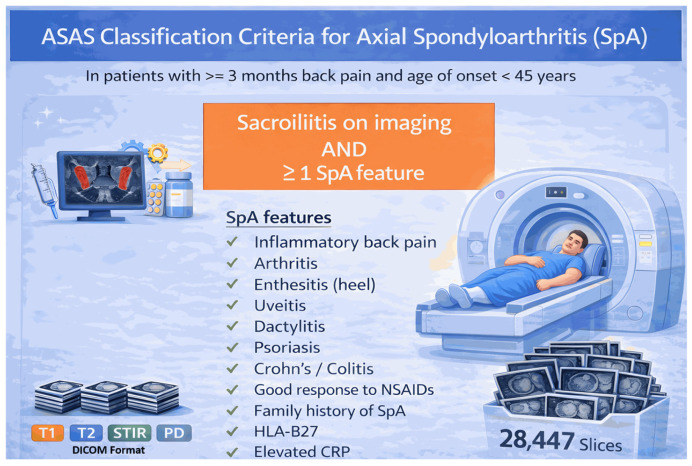
ASAS classification criteria [25]. (Visual design prepared using ChatGPT 5.4 (OpenAI, San Francisco, CA, USA).)

In this study, SIJ MRIs of cases evaluated within the axSpA diagnostic spectrum are analysed alongside rheumatological, clinical, and laboratory findings. Patient selection was performed in accordance with the Assessment of SpondyloArthritis International Society (ASAS) axial SpA classification criteria, as shown in Figure 2. In this context, based on the imaging arm of the ASAS criteria, cases with sacroiliitis findings on SIJ MRI according to the ASAS definition and at least one SpA feature was included in the study as the axSpA group, creating a clinically homogeneous and diagnostically meaningful patient population. The CBP control group (non-axSpA) comprised participants who had presented to the rheumatology department with back pain for at least 3 months and consequently underwent SIJ MRI. Participants in this group were cases that were not evaluated in favour of axSpA based on clinical and/or imaging findings and did not meet the ASAS axial SpA classification criteria. Inclusion and exclusion criteria for the dataset and group definitions are presented in Supplementary Section S1.

Within the scope of the study, a total of 367 participants were analysed, comprising an axSpA group (*n* = 193) and a non-axSpA CBP control group (*n* = 174), using data obtained between January 2020 and December 2022. The demographic and clinical characteristics of the participants are summarised in Table 1.

**Figure 3:**
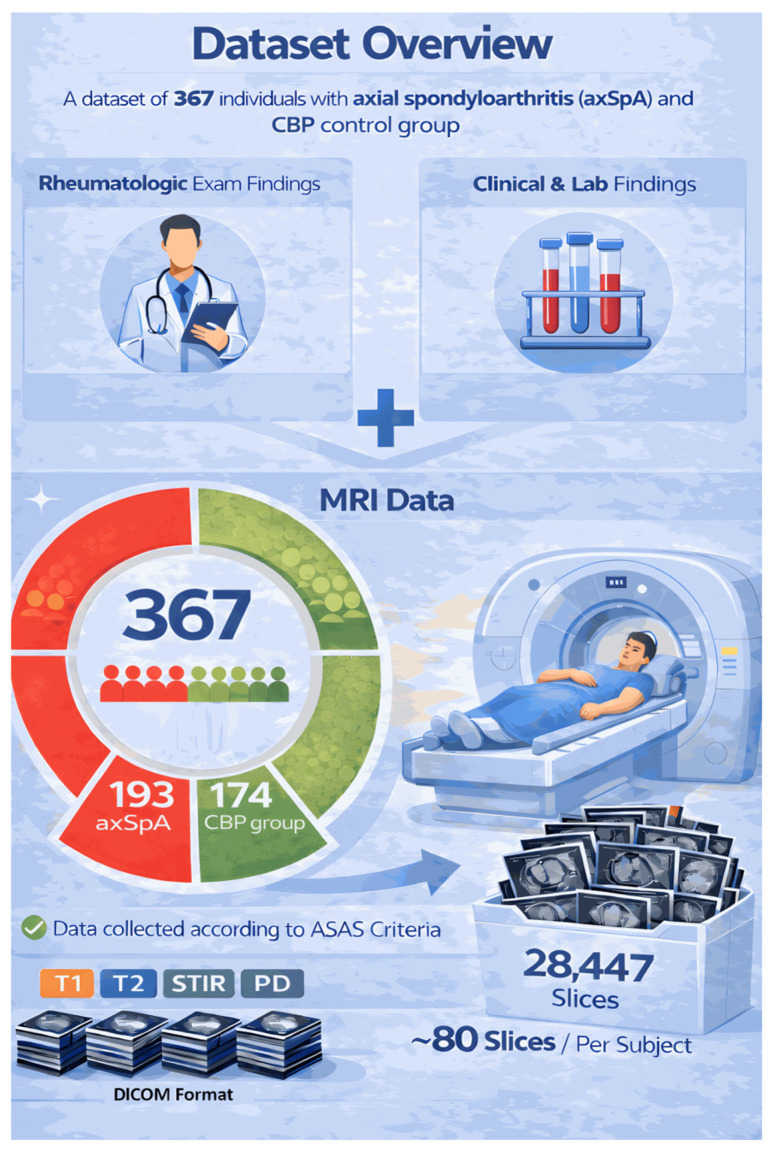
General structure of the dataset. (Visual design prepared using ChatGPT 5.4 (OpenAI, San Francisco, CA, USA).)

The overall structure of the data set is shown in Figure 3.

During the preparation of Figure 2 and Figure 3, the generative artificial intelligence tool ChatGPT 5.4 (OpenAI, San Francisco, CA, USA) was used to support the visual design and layout of the figures. The scientific content and final verification of these figures were performed by the authors.

In addition to SIJ MRIs, the study included rheumatological, clinical, and laboratory data for each patient, such as inflammatory back pain (IBP), chronic back pain (CBP), arthritis, enthesitis, uveitis, dactylitis, psoriasis, Crohn’s disease/ulcerative colitis, good response to non-steroidal anti-inflammatory drugs (NSAIDs), family history of SpA, HLA-B27 positivity, and elevated C-reactive protein (CRP) levels. These clinical and laboratory variables were used as a structured dataset in CSV format to be integrated into the classification process; binary (presence/absence) clinical variables were coded as 0 and 1.

### 2.3. MRI Acquisition Protocol

SIJ MRIs of all participants included in the study were acquired using a Siemens Magnetom Aera MRI scanner (Erlangen, Germany) with a 1.5 T magnetic field strength under standard protocols. The acquired MRIs were comprehensively evaluated and labelled by two experienced rheumatologists (28 years and 12 years of experience) and one radiologist (8 years of experience). In accordance with the diagnoses established by mutual agreement between the radiologists and rheumatologists, multiple MRI sequences belonging to a total of 367 participants were recorded in DICOM format. Each sequence contained an average of 20 two-dimensional slices per participant, corresponding to approximately 80 slices per participant. The total number of slices used in the study was 28,447.

### 2.4. MRI Sequences

**Figure 4:**
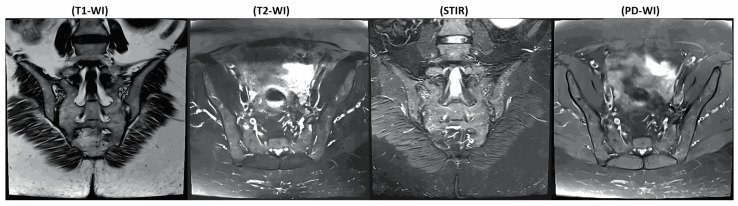
Representative T1-WI, T2-WI, STIR, and PD-WI MRI sequences of the sacroiliac joint from the same participant.

As shown in Figure 4, four different MRI sequences were used in this study. The literature reports that the combined analysis of different MRI sequences can increase diagnostic accuracy [26,27]. In this context, T1-weighted images allow for the evaluation of anatomical structures and structural changes, while T2-weighted sequences enable inflammation and oedema to be visualised more clearly [28,29]. STIR sequences have been used to demonstrate BME and active inflammation, while PD-weighted sequences have been used to evaluate joint surfaces and soft tissues with high contrast. The combined analysis of these sequences enables a complementary evaluation of active and structural pathologies in the SIJs.

### 2.5. Data Preparation and Model Training Framework

The SIJ MRIs obtained from participants in DICOM format were converted into two-dimensional PNG slices as part of data standardisation; the original slices and binary masks were rescaled to 256 × 256 dimensions. The dataset was divided into training, validation and test subsets on a patient-by-patient basis. Twenty percent of the dataset was randomly selected and set aside as the test set; the remaining 80% was structured into a 64% training and 16% validation subset. To prevent data leakage, all slices belonging to each patient were included in only one dataset. Density normalisation and data augmentation were applied. The model training processes were performed on GPU-supported workstations. Detailed technical information regarding data preprocessing and model training processes is provided in Supplementary Section S2.

### 2.6. Region of Interest (ROI)

**Figure 5:**
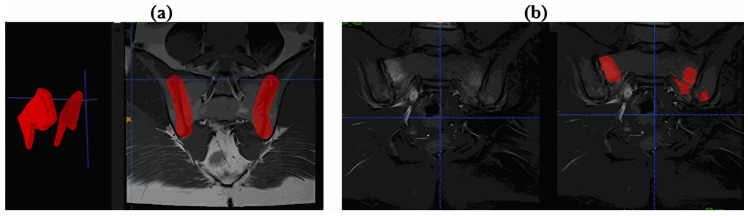
(**a**) Three-dimensional (**left**) and two-dimensional cross-sectional (**right**) representation of SIJ mask images, (**b**) masks showing active inflammatory regions created in ITK-SNAP for segmentation.

In this study, masks used in classification and segmentation processes were created separately to serve different methodological purposes. In classification models, SIJ regions were presented to the model using the ROI approach to focus on clinically meaningful anatomical regions. SIJ masks were created manually and semi-automatically by experts on two-dimensional MRI slices using ITK-SNAP [30] (v4.4.0) software; slice-to-slice label continuity was ensured using ITK-SNAP’s Interpolate Labels feature. The three-dimensional view of the SIJ masks and the mask overlay on a single slice are presented in Figure 5a.

The masks used in the segmentation models were created using the same software with a semi-automatic active contour (balloon) approach to visualise inflammation-related active regions. In this process, the initial balloons placed on oedematous regions were optimised through iterative expansion; manual corrections were applied where necessary to improve adaptation to lesion boundaries. The resulting pixel-level segmentation masks represent the active inflammatory regions and are shown in Figure 5b.

## 3. Proposed Methodology

This study proposes an explainable comprehensive CDSS that combines multi-sequence MRI data with rheumatological, clinical, and laboratory findings at the patient level for the early diagnosis of axSpA. The system begins with preprocessing and ROI steps applied to images obtained from T1-WI, T2-WI, STIR, and PD-WI weighted MRI sequences. The images are analysed using separately trained DL-based models for each MRI sequence; slice-level probability scores are combined using a dynamic Top-K averaging strategy to obtain a single axSpA probability score at the patient level (Part 1). Additionally, a clinical risk score (Part 2) is calculated using an ML-based method incorporating rheumatological, clinical, and laboratory findings.

**Figure 6:**
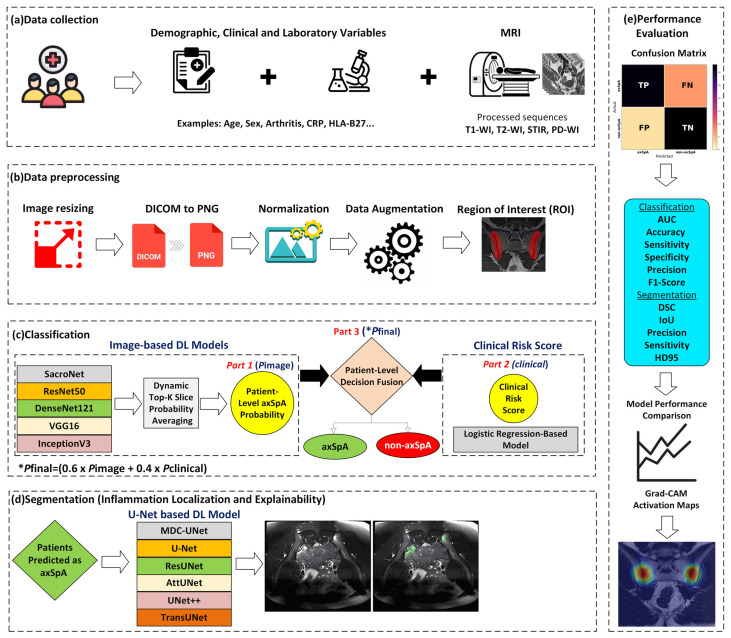
Proposed comprehensive CDSS workflow for axSpA. (**a**) Demographic/clinical/laboratory data and multi-sequence MRI (T1-WI, T2-WI, STIR, PD-WI) were collected. (**b**) Data underwent preprocessing; the SIJ region was defined as an ROI in the MRI. (**c**) Patient-level axSpA probability was generated from image-based DL models; combined at the decision level with a logistic regression-based clinical risk score to obtain *P_final_*_._ (**d**) U-Net-based segmentation was applied for localisation and interpretability of inflammation in cases classified as axSpA. (**e**) Classification and segmentation performance were evaluated using standard metrics; interpretability was supported by Grad-CAM visualisations.

Image-based axSpA probability outputs are combined with the clinical risk score using a patient-level decision integration approach (Part 3) to form the final diagnostic decision. In patients with a predicted axSpA diagnosis, U-Net-based deep learning models are used to visualise potential active regions associated with inflammation, thereby enhancing clinical interpretability. The general workflow of the proposed system is shown in Figure 6.

### 3.1. Multi-Sequence Image-Based DL Framework (Part 1)

Since axSpA -related T1-WI, T2-WI, STIR, and PD-WI MRI sequences reflect different tissue and pathological features, the sequences were treated independently, and separate DL models were trained for each sequence. In this context, CNN-based architectures commonly used in the literature, such as ImageNet pre-trained ResNet50, DenseNet121, VGG16, and InceptionV3, were adapted and used for MRI data.

Additionally, the SacroNet model, developed specifically for axSpA, was also included in the study. SacroNet is a ROI-guided, low-parameter (~110 K) CNN architecture focused on the SIJ region, trained without using a pre-trained backbone. The model aims to effectively learn axSpA-specific inflammatory and structural patterns; thanks to its lightweight architectural design, it aims to produce more stable predictions at the patient level by reducing overfitting under limited data conditions.

#### Image-Based Patient-Level Decision Strategy of DL Models (Dynamic Top-K Averaging)

During the testing phase, all MRI slices belonging to the patient were evaluated by the DL model, producing probability scores at the slice level. Based on the assumption that not all slices contribute equally to the diagnosis, the Top-K approach was adopted when making patient-level decisions. Accordingly, slice-based probability scores were sorted from highest to lowest, and K slices were dynamically selected from among the slices with the highest probability, with a minimum of 5 slices and a maximum of half the total number of slices. The arithmetic mean of the probability scores of the selected Top-K slices was taken to obtain the sequence-based classification score at the patient level (Equation (1)).

(1) $$P_{patient}^{\left(s\right)}=\frac{1}{K}\sum_{i=1}^{K}P_{i}^{\left(s\right)}$$

For example, when there is a total of 20 slices for a sequence belonging to a patient, the model generates a probability score for each slice, and these scores are sorted from highest to lowest. Since K in Dynamic Top-K Averaging is determined to be at least 5 and not to exceed half the total number of slices, K can be at most 10 in this example. In this case, the Top-K slice with the highest probability (e.g., K = 10) is selected, and the arithmetic mean of the probability scores of these slices is taken to obtain a single probability score at the patient level.

This approach provides a consistent decision mechanism for patients with different numbers of slices and offers a more representative patient-level assessment by reducing the impact of the initial and final slices, where pathological findings are limited, on the decision.

### 3.2. Clinical Risk Score Estimation via ML (Part 2)

The Clinical Risk Score was calculated using a logistic regression (LR)-based ML approach, utilising rheumatological, clinical, and laboratory findings. LR was preferred because it allows the contribution of clinical variables to axSpA risk to be presented in a clear and interpretable manner via odds ratio (OR). Due to the relatively limited sample size of the clinical dataset, a more transparent and generalisable approach was adopted instead of high-parameter and complex ML models. The Clinical Risk Score was used not as an independent diagnostic model producing a probability output in the range of 0–1 for each patient, but as a clinical decision component supporting image-based DL models.

### 3.3. Patient-Level Decision Fusion Strategy (Part 3)

The patient-level probability outputs obtained from image-based DL models and the Clinical Risk Score are combined using a weighted linear fusion ($P_{final}$) approach to support the patient-level diagnostic decision. Image-based probabilities are weighted at 0.60, while the Clinical Risk Score is weighted at 0.40. These weighting coefficients were determined by systematically testing different combinations (w ∈ [0,1]) on the validation set.

ROC analysis was performed on the composite score obtained for each w value; threshold-sensitive metrics such as the Youden index (J = sensitivity + specificity − 1) and F1-score were evaluated together. When comparing only image outputs with fusion outputs, it was observed that adding the clinical risk score reduced false negatives, particularly in cases close to the decision threshold, and improved balanced accuracy and F1-score metrics. The weight selection was determined as the combination that optimised these metrics in the most balanced way in the validation set, with the aim of reducing false negatives while maintaining sensitivity in early diagnosis. As a result of this evaluation, the combination $w_{image}$ = 0.60 and $w_{clinical}$ = 0.40, which provided the most balanced performance in the validation set, was selected.

The combined patient-level axSpA probability is calculated as in Equation (2):

(2) $$P_{final}=(0.6\times P_{image}+0.4\times P_{clinical})$$

$P_{final}$ was converted to binary classification based on the optimal threshold determined using ROC analysis, and an axSpA or non- axSpA decision was generated for each patient.

### 3.4. U-Net–Based Active Inflammation Segmentation Within SIJ Regions (Part 4)

In patients with suspected axSpA diagnosis, U-Net-based DL models were utilised to visually present active inflammation of the SIJs. The segmentation process proposed in this study was designed not as a mechanism that directly provides the final diagnosis decision, but as a decision support component that supports radiological evaluation, increases interpretability, and provides the clinician with image-based objective findings.

## 4. Experimental Results

This section presents the experimental results of the study. The classification performance of DL models at the patient level, clinical risk score results, and the final diagnostic outputs obtained by combining these two components at the decision level are reported. Additionally, segmentation results from U-Net-based DL models showing active inflammation in patients predicted to have axSpA are presented through representative images.

### 4.1. Evaluation Metrics

The performance evaluations of classification models were analysed using the metrics of accuracy, sensitivity, specificity, precision, F1 score, and area under the curve (AUC). Additionally, a confusion matrix was reported to demonstrate the relationship between model predictions and actual labels. All metrics were calculated at the patient level and used in comparisons between models. For the performance evaluation of segmentation models, the dice similarity coefficient (DSC), intersection over union (IoU), precision, sensitivity, and 95% hausdorff distance (HD95) metrics were used. The formulas for these metrics are provided in Supplementary Tables S3–S5.

To increase statistical rigour, 95% confidence intervals (CI) for the baseline classification (AUC) and segmentation (DSC) outputs were obtained using non-parametric bootstrap resampling (B = 1000 resamples) on separate test sets.

### 4.2. Patient-Level Performance of the Proposed Image-Based DL Framework (Part 1)

This section presents the patient-based diagnostic performance of DL models in the early diagnosis of axSpA, separately based on T1-WI, T2-WI, STIR, and PD-WI MRI sequences. In this context, the results for the T1-WI MRI sequence are shown in Table 2.

When examining the patient-level diagnostic performance results for the T1-WI sequence, it is observed that the VGG16 model provides the highest discriminative power (AUC = 0.920) and accuracy (Accuracy = 0.878) values. The DenseNet121 and InceptionV3 models, on the other hand, demonstrated more balanced performance in terms of sensitivity and specificity, yielding stable results. The SacroNet model, which has fewer parameters (~110 K), performed successfully in correctly identifying axSpA cases, particularly with its high sensitivity value (Sensitivity = 0.897). The ResNet50 model was competitive in terms of all metrics but showed more limited discriminatory performance compared to other models.

**Figure 7:**
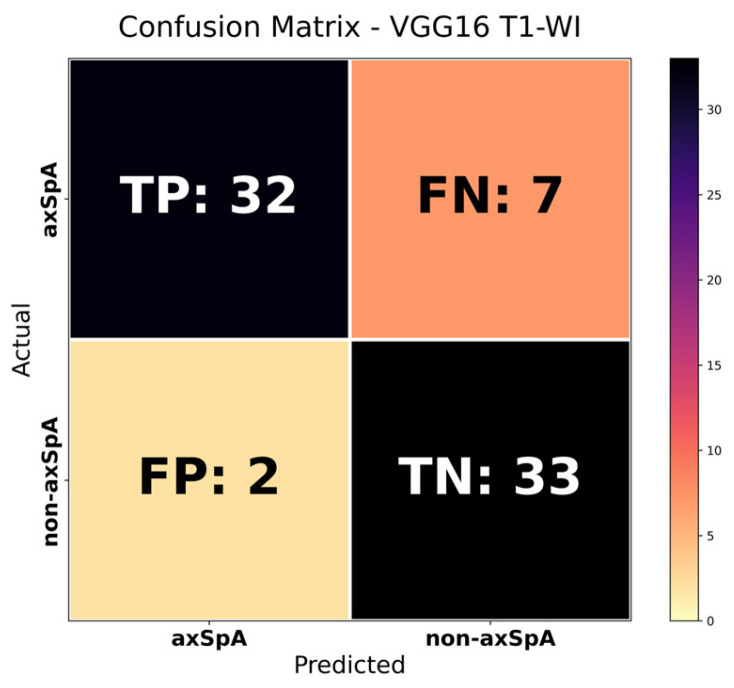
VGG16 T1-WI confusion matrix.

**Figure 8:**
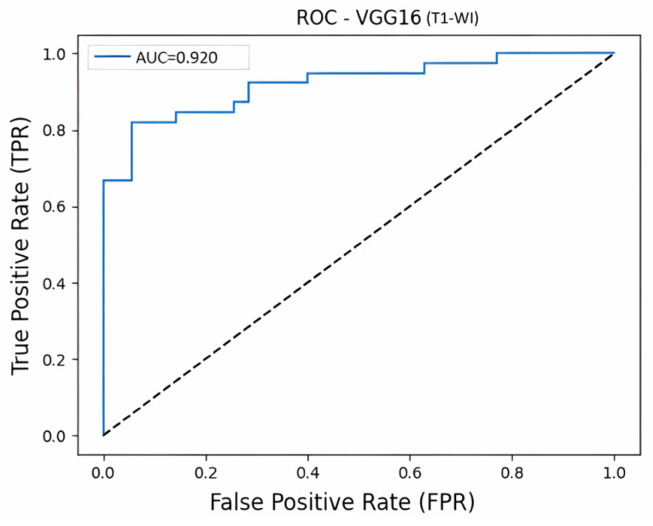
VGG16 T1-WI ROC curve.

When the findings are evaluated together, it is seen that the VGG16 model contributes significantly to reducing false positive classifications with its high specificity (Specificity = 0.942) and precision (Precision = 0.941) values. The confusion matrix for the VGG16 model, which showed the highest discriminatory performance in the T1-WI sequence, is presented in Figure 7, and the ROC curve is presented in Figure 8.

Patient-level diagnostic performance results for T2-WI MRI sequences are shown in Table 3.

Table 3 shows that the SacroNet model performs better than other models in terms of discriminative power (AUC = 0.717). However, despite its high specificity and accuracy values, the SacroNet model may miss some axSpA cases due to its low sensitivity. The DenseNet121 model, on the other hand, offers more balanced discrimination in terms of sensitivity and F1-Score. The ResNet50, VGG16, and InceptionV3 models showed moderate classification success.

When the findings are evaluated together, while the SacroNet model stands out in terms of overall discriminative power, it is considered that the balanced performance of the DenseNet121 model in terms of sensitivity and F1-Score may offer a more reliable classification profile for CDSS. The confusion matrix for the DenseNet121 model, which showed the best performance in the T2-WI sequence, is presented in Supplementary Figure S1, and the ROC curve is presented in Supplementary Figure S2.

The patient-level diagnostic performance results for the STIR MRI sequences are shown in Table 4.

Upon examining Table 4, it is observed that the DenseNet121 model demonstrates the highest performance in terms of discrimination (AUC = 0.793) and accuracy (Accuracy = 0.771) metrics. The SacroNet model, on the other hand, produced successful results in detecting axSpA cases with a high sensitivity value (Sensitivity = 0.815) but its lower specificity value led to an increase in false positive classifications. The ResNet50 model limited false positive classifications with high specificity (Specificity = 0.875) and precision (Precision = 0.826) values but may miss some axSpA cases due to its low sensitivity value. The VGG16 and InceptionV3 models, on the other hand, offered balanced performance metrics but more limited results compared to the DenseNet121 model.

When the findings are evaluated together, it is considered that the DenseNet121 model offers the strongest performance in terms of discriminative power and classification balance in the STIR sequence and may provide a more reliable classification profile for CDSS applications. The confusion matrix for the DenseNet121 model, which showed the best performance in the STIR sequence, is presented in Supplementary Figure S3, and the ROC curve is presented in Supplementary Figure S4.

The patient-level diagnostic performance results for PD-WI MRI sequences are shown in Table 5.

Upon examining Table 5, it is observed that the VGG16 model demonstrates the strongest performance in terms of the metrics of discriminative power (AUC = 0.765), accuracy (Accuracy = 0.750), and F1-Score (0.810). The VGG16 model also achieved successful results in detecting axSpA cases with a high sensitivity value (Sensitivity = 0.909), but the relatively low specificity value led to an increase in false positive classifications. The SacroNet and DenseNet121 models offered more balanced performance in terms of sensitivity and F1-Score, while the ResNet50 model limited false positive classifications with high specificity (Specificity = 0.82) and precision (Precision = 0.800) values. The InceptionV3 model, on the other hand, showed balanced performance metrics but yielded more limited results compared to the VGG16 model.

When the findings are evaluated together, it is considered that the VGG16 model stands out in terms of discriminative power and sensitivity in the PD-WI sequence; however, due to its limited specificity, false positive results in CDSS should be carefully evaluated. The confusion matrix for the VGG16 model, which showed the best performance in the PD-WI sequence, is presented in Supplementary Figure S5, and the ROC curve is presented in Supplementary Figure S6.

When all MR sequences and DL models are evaluated together, the VGG16 model trained on T1-WI images is seen to offer the highest discriminatory performance in axSpA classification and the most consistent classification results at the patient level (Table 6).

**Figure 9:**
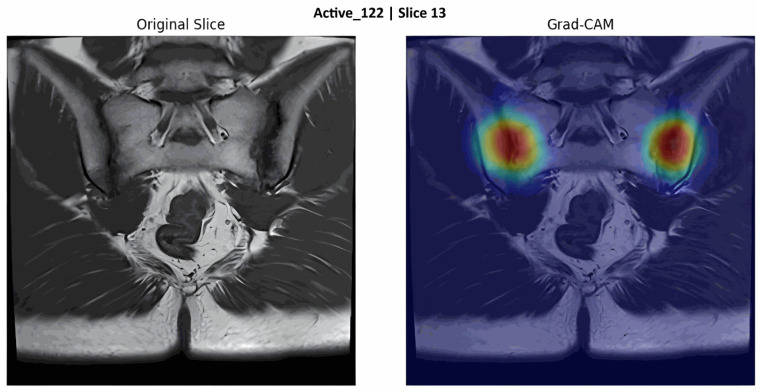
Grad-CAM activation map output. (The colors indicate the intensity of model attention in the Grad-CAM activation map. Warmer colors (red/yellow) represent higher attention, while cooler colors (blue/green) represent lower attention.)

These findings suggest that the T1-WI sequence offers a powerful imaging characteristic for representing structural changes and that the VGG16 architecture can effectively learn these structural features. Grad-CAM analysis was applied to visualise the anatomical regions on which the relevant model focused during the decision process. A representative Grad-CAM activation map output showing activation areas concentrated in the SIJ region in a patient predicted to have axSpA is presented in Figure 9.

#### Multimodal Decision Fusion Strategy

In the multimodal approach, based on the assumption that different MRI sequences provide complementary diagnostic information, the probability outputs of sequence-based DL models at the patient level have been combined at the decision level. In this context, the models that provided the best performance at the patient level were selected for each sequence; VGG16 was used for T1-WI and PD-WI images, and DenseNet121 was used for STIR and T2-WI sequences. The multimodal probability score ($P_{multi}$) was calculated using weights determined according to the AUC values reflecting the discriminative performance of the selected sequence models and normalised to sum to 1. The multimodal probability score is shown in Equation (3):

(3) $$P_{multi}=0.293\times P_{T1}+0.212\times P_{T2}+0.252\times P_{STIR}+0.243\times P_{PD}$$

**Figure 10:**
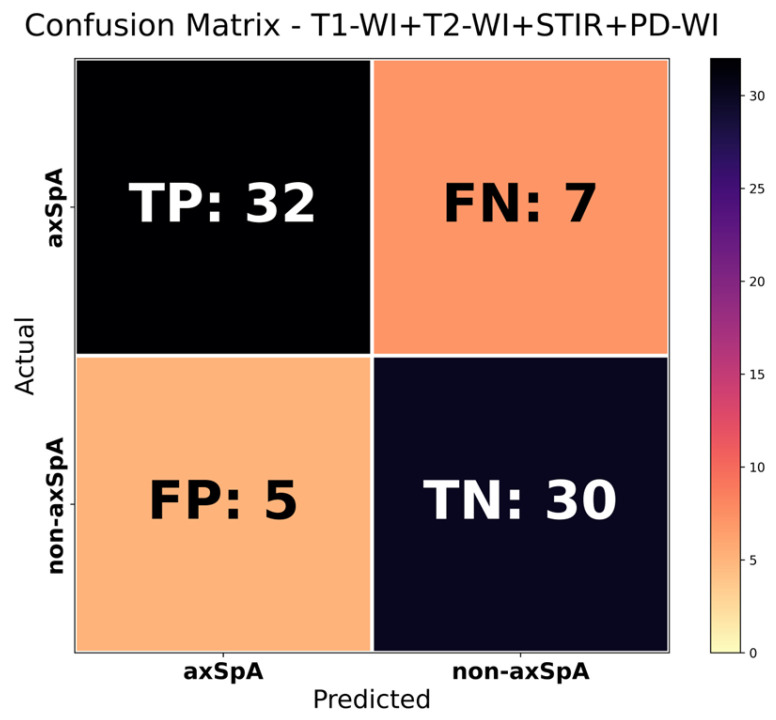
Multimodal confusion matrix.

**Figure 11:**
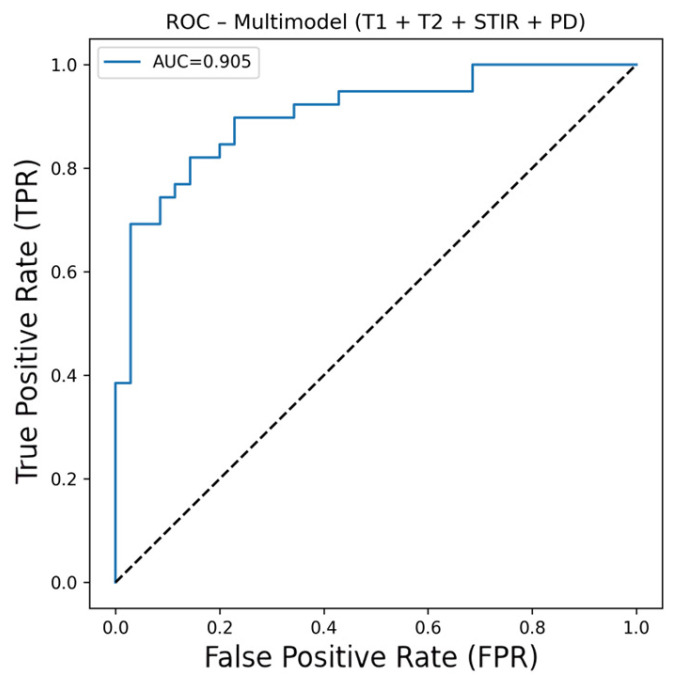
Multimodal ROC curve.

$P_{multi}$ was converted to binary classification using the defined threshold value (0.70). If not all MRI sequences were available for a patient, only the probability scores for the existing sequences were used, and the corresponding weights were renormalised to sum to 1 to calculate $P_{multi}$. The patient-level performance results for the multimodal are presented in Table 7, the confusion matrix in Figure 10, and the ROC curve in Figure 11.

The multimodal approach aimed to reflect the multi-sequence evaluation logic in clinical practice, as demonstrated by the patient-level fusion score (AUC = 0.905, accuracy = 0.837, sensitivity = 0.820, F1 = 0.842) compared to individual sequence models. The VGG16 model trained on T1-WI (AUC = 0.920, accuracy = 0.878, sensitivity = 0.820, F1 = 0.876), which demonstrated the highest and most consistent performance among all models, appears superior to the multimodal in terms of general performance metrics, particularly AUC and accuracy. Therefore, the T1-WI VGG16 model was adopted as the primary model in this study. The multimodal was reported as a secondary/auxiliary analysis to evaluate the complementary contribution of the sequences. Furthermore, in real-world conditions, it may not always be possible to obtain all MRI sequences completely for every patient. Therefore, it is anticipated that when the T1-WI sequence is available, performing classification primarily through the T1-WI-based model will increase practical applicability; when other sequences are available, the complementary contribution provided by multimodal fusion may support the confidence of the diagnostic decision.

### 4.3. Patient-Level Performance of the Proposed ML-Based Clinical Risk Score (Part 2)

The clinical risk score model was evaluated at the patient level, and the contributions of clinical variables to axSpA diagnosis were analysed using logistic regression-based odds ratios (Table 8). The results show that inflammatory back pain (OR = 7.612) and elevated CRP (OR = 5.361) contributed most strongly to the clinical risk score. HLA-B27 positivity (OR = 2.655) and arthritis (OR = 1.984) provided moderate discriminatory effects, while the contribution of other clinical variables was limited.

The CBP variable was not included in the clinical risk score calculation, as it is a common symptom reflecting the reason for presenting to the clinic in both the axSpA and non-axSpA control groups and was not expected to provide a discriminatory contribution between the groups. Accordingly, the OR analyses reported in Table 8 were performed using clinical variables defined excluding CBP.

These findings suggest that rather than using the clinical risk score as an independent diagnostic model, it may be more appropriate to position it as a complementary decision component alongside the outputs of image-based DL models.

### 4.4. Patient-Level Performance of the Proposed Decision Fusion Framework (Part 3)

This section presents the results of integrating the clinical risk score at the patient level with the VGG16 model trained on T1-WI images, which demonstrated the highest performance. The ROC analysis performed at the patient level resulted in an optimal decision threshold of τ = 0.8166 for the T1-WI VGG16 model, based on the Youden index. However, a more balanced threshold value of τ = 0.70 was preferred for the final classification decisions, to leverage the supportive role of the clinical risk score and preserve sensitivity in early diagnosis.

Similar ROC-based threshold determination approaches at the patient level have been used in the literature; In the JointNet model, to ensure that active inflammation is not overlooked, a threshold value of 0.6 defined in the range (−1,1) corresponds to approximately 0.8 on a normalised scale [31], while in the study by Xie et al., the optimal threshold value of 0.637 was determined based on the Youden index [32]. These findings indicate that the threshold value of τ = 0.70 used in our study is consistent with the literature.

Image-based probabilities ($P_{image}$) and clinical risk scores ($P_{clinical}$) were converted to the final diagnosis probability ($P_{final}$) through weighted linear combination at the decision level; it was demonstrated that the clinical risk score increases diagnostic confidence by reducing false negatives, particularly in borderline cases close to the decision threshold. Results from selected representative cases demonstrating the patient-level impact of the fusion approach are presented in Table 9.

### 4.5. Segmentation Performance of the Proposed U-Net-Based Active Inflammation Model (Part 4)

In this study, U-Net-based segmentation models were used to visually demonstrate the distribution of active inflammation within the SIJ region in patients classified as axSpA and to enhance the interpretability of the decision-making process. The segmentation component was designed not as a mechanism that directly produces the final diagnostic decision, but as a decision support layer that provides image-based objective findings regarding the location and extent of the lesion, thereby supporting radiological evaluation.

#### 4.5.1. Comparative Experimental Setup and Proposed MDC-UNet Model

Active inflammation regions at the SIJ level exhibit a small, fragmented, and ill-defined structure, particularly in early-stage cases. These morphological challenges expose segmentation models to two fundamental errors: (1) failure to detect oedema areas (under-segmentation) due to background dominance and (2) over-segmentation in low-contrast transition regions.

The multi-scale dilated contextual U-Net (MDC-UNet) developed in this study preserves the encoder–decoder skeleton of the classic U-Net [33] while being customised to adapt to the variable scale and distribution of oedema. The architecture integrates multi-scale dilated structures and attention architectures on the encoder side [34,35]. This hybrid structure aims to increase sensitivity to small lesions and optimise boundary placement.

To validate the effectiveness of the proposed MDC-UNet architecture, comparative experiments were conducted with models commonly used in the literature and representing different architectural paradigms. In this context, the U-Net [33], ResUNet [36], Attention U-Net [34], UNet++ [37], and TransUNet [35] models were used as reference methods.

The quantitative segmentation performance of the proposed MDC-UNet and comparative models on the test sets of T2-WI and STIR sequences is summarised in Table 10 and Table 11, respectively. The primary reason for preferring T2-WI and STIR sequences in segmentation analyses is that these sequences can reveal BME and active inflammation with high sensitivity.

When analysing the T2-WI sequence, the proposed MDC-UNet model achieved the highest Dice coefficient of 0.682 (95% CI: 0.655–0.708), IoU (0.646), and Precision (0.713) values. Furthermore, by obtaining the lowest HD95 value (26.21 pixels), it demonstrated more accurate geometric precision in determining inflammation boundaries compared to other models. Although the UNet++ model had the highest sensitivity value (0.852), its lower Precision value indicates a tendency for the model to produce false positives.

When examining the STIR sequence results, the MDC-UNet model demonstrated superior performance similar to the T2-WI sequence. The model stood out with the highest Dice coefficient of 0.751 (95% CI: 0.726–0.776), IoU (0.716), and lowest HD95 distance (19.25 pixels). Another noteworthy finding is that the standard deviation values of MDC-UNet (±0.021 for DSC, ±0.86 for HD95) were significantly lower than those of other models. This demonstrates that the proposed architecture produces stable and robust results under different conditions.

#### 4.5.2. Qualitative Evaluation

**Figure 12:**
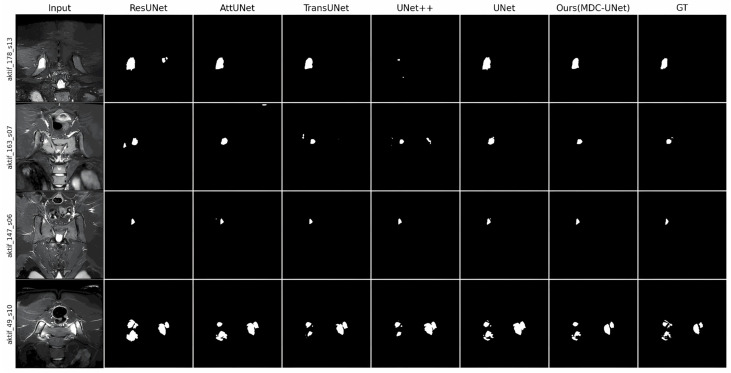
Qualitative analysis of segmentation outputs from the proposed MDC-UNet and state-of-the-art models on the STIR sequence.

**Figure 13:**
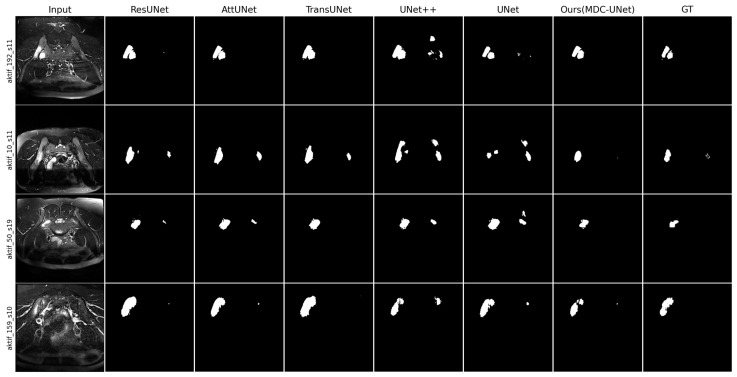
Qualitative analysis of segmentation outputs from the proposed MDC-UNet and state-of-the-art models on T2-WI sequences.

The visual segmentation outputs of the models for STIR and T2-WI sequences are presented in Figure 12 and Figure 13, respectively. Upon examining the visual outputs, it was observed that the basic U-Net and ResUNet models exhibit boundary over-segmentation in low-contrast regions or missed small oedema foci. In contrast, MDC-UNet architecture, thanks to its multi-scale context inference mechanism, more effectively characterized the complex topology of inflammation. This approach enables the segmentation of thin oedema lines adjacent to the joint space with high fidelity to the Ground Truth (GT).

**Figure 14:**
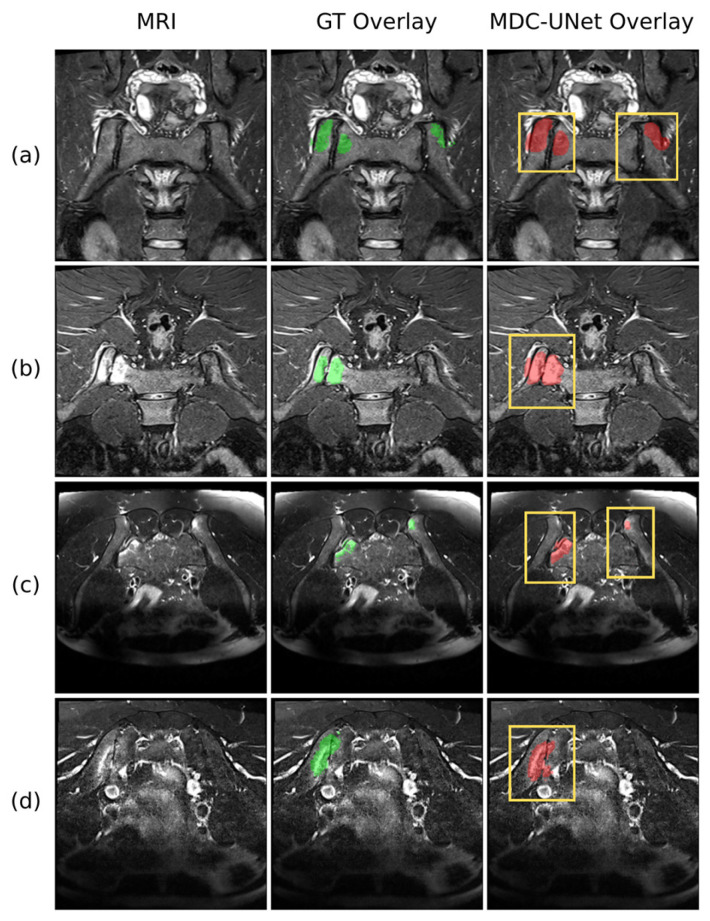
Qualitative segmentation results of the proposed MDC-UNet across varying anatomical configurations and MRI sequence types. The original MRI, ground truth overlay (green), and model prediction overlay (red) are shown column-wise, with yellow bounding boxes indicating the sacroiliac joint regions of interest. Rows (**a**,**b**) represent STIR sequences with bilateral and unilateral oedema involvement; rows (**c**,**d**) represent T2-WI sequences where oedema contrast is comparatively reduced.

The segmentation performance of the proposed MDC-UNet model on different MR sequences and varying lesion morphologies is presented in Figure 14. The visual outputs demonstrate that the model (red mask) achieves high spatial overlap with the GT (green mask) delineated by the expert radiologist.

Particularly in the examples from the STIR sequence (Rows a and b), it was observed that the model successfully distinguished the boundaries between the bright signal intensity of the oedema and the background. In T2-WI sections with more complex anatomical details (Rows c and d), it is noteworthy that MDC-UNet performs accurate detection while preserving boundary delineation without falling into the over-segmentation error. The model’s stable performance in both bilateral and unilateral lesions supports the high generalisation ability of the developed architecture against clinical variations.

## 5. Discussion

The findings of this study demonstrate that MRI sequence selection and patient-level decision integration strategies significantly affect model performance for axSpA diagnosis.

A significant portion of MRI-based DL studies have focused on specific sequences or used limited sequence combinations (Supplementary Tables S1 and S2). Accordingly, modelling and comparing the four sequences separately allowed for the evaluation of how the different components of SIJ pathology are reflected in model performance.

The highest diagnosis performance among DL models (AUC = 0.920) was obtained from the VGG16 model on the T1-WI sequence. The higher classification success of the T1-WI sequence may be due to its ability to reveal chronic structural changes with high anatomical contrast. STIR and T2-WI sequences are clinically critical for assessing active inflammation. Although the heterogeneous and time-dependent distribution of inflammatory signal increases in these sequences may limit model performance, they play a complementary role in determining disease activity. It is thought that the PD-WI sequence may produce more variable results in terms of performance across models due to heterogeneity and variation in signal distribution. In the multimodal approach, when the patient-level probability outputs of sequence-based DL models are combined based on the assumption that sequences provide complementary information, it is observed that the multimodal model (AUC = 0.905) cannot match the performance of the T1-WI-based VGG16 (AUC = 0.920). Therefore, T1-WI VGG16 was preferred as the primary image-based classification model in the study. Multimodal fusion was reported as a secondary/auxiliary analysis to demonstrate the complementary contribution of sequences and to produce a supportive decision score in cases where multiple sequences were available. Given that sequence deficiencies are possible in real-world clinical settings, initiating the decision primarily through this model when T1-WI is available may increase practical applicability; in cases where additional sequences are present, multimodal fusion can complementarily support diagnostic confidence.

Furthermore, the proposed SacroNet architecture developed in this study demonstrated stable and competitive performance even with limited datasets, thanks to its efficient parameter structure. It is considered that lightweight models may be a more suitable option for CDSS applications due to the potential of the optimised architectural design to reduce overfitting risk and computational cost. Although SacroNet offers a more compact architectural profile compared to transfer learning-based backbone models such as VGG16/ResNet50/DenseNet121/InceptionV3, it can be positioned as an alternative that can be evaluated in terms of performance-cost balance, thanks to its acceptable diagnostic performance at the patient level.

While a significant portion of the literature reports performance at the slice level, clinical diagnosis is provided at the patient level. The proposed Dynamic Top-K Averaging approach in this study increased decision consistency by combining slice predictions at the patient level, and results closer to clinical assessment were observed. This approach reduces noise effects and contributes to balancing false positive/negative decisions by focusing on slices with more diagnostically distinct patterns rather than evaluating all slices equally. Thus, the model outputs are seen to have a structure more compatible with the clinical decision-making process.

Recent studies have shown that evaluating MRI-based DL outputs alongside clinical variables can improve diagnostic accuracy [32,38]. In this study, the integration of the LR-based clinical risk score with image-based probability scores contributed to increased diagnostic confidence, particularly in borderline cases. This approach not only improved quantitative performance metrics but also provided a comprehensive evaluation framework that more realistically reflects the multidimensional nature of the clinical decision-making process.

Supporting classification outputs with visual evidence is critical for clinical reliability and physician acceptance. In this context, the developed MDC-UNet segmentation model demonstrated more consistent performance than U-Net derivatives commonly used in the literature, particularly in identifying small, fragmented, and irregular inflammation foci. It is considered that the multi-scale extended convolution structure may have contributed to the representation of inflammatory patterns of different sizes; particularly by helping to preserve contextual integrity in low-contrast and ill-defined areas, it may have provided a more balanced segmentation performance. The low Hausdorff distance values obtained suggest that inflammation boundaries can be determined with greater accuracy relative to Ground Truth, indicating that the model may offer advantages not only in terms of quantitative accuracy but also anatomical precision.

A significant portion of the reported delay in diagnosing axSpA may be related to the possibility of early-stage non-specific back pain symptoms being confused with other mechanical causes and to delays in decisions to refer patients for advanced imaging. The proposed CDSS framework presented in this study offers a framework that could assist in early risk classification, particularly in non-rheumatology clinical settings (e.g., primary care services or general orthopaedic clinics), and support the earlier referral of high-risk patients for specialist evaluation. Furthermore, it is considered that patient-level decision integration and visually supported segmentation outputs could support clinician confidence and contribute to the decision-making process by reducing the uncertainty accompanying clinical decisions. The proposed CDSS has the potential to contribute to reducing diagnostic delays when appropriate clinical integration is achieved. In this context, the key strengths of the study can be summarised as follows.

### Strengths of the Study

(i) Systematic comparison of models developed on multiple MRI sequences at the patient level, (ii) Integration closer to clinical decision-making by transferring slice outputs to the patient level using the Dynamic Top-K Averaging strategy, (iii) Combining image-based probabilities with an LR-based clinical risk score to form the final diagnostic decision, (iv) Evaluating the SacroNet model as an alternative to common transfer learning-based backbones due to its parameter-efficient and lightweight architecture, and (v) Supporting interpretability through the visualisation of small/fragmented inflammation foci using the MDC-UNet developed in this study. This holistic CRS framework offers a clinically transferable approach by integrating these components, which are often addressed separately in the literature, under a single structure.

## 6. Conclusions and Future Work

This study, developed to support the early diagnosis of axSpA, presents a comprehensive DSS approach that combines multi-sequence MRI data at the patient level, integrates clinical and laboratory risk factors into the decision-making process, and aims to enhance clinical interpretability with segmentation-supported explainability. The findings indicate that sequence-based model performance differences, patient-level decision integration strategies, and clinical risk scores play a complementary and decisive role in diagnostic accuracy.

The single-centre and retrospective nature of the data ensures standardised protocols and labelling consistency but also necessitates multicentre/external validation for generalisability. However, the study design, which did not involve random sampling, the absence of a blinded evaluation protocol in the labelling/assessment processes, and the fact that the sample represents a specific patient population should be considered factors that may limit the generalisability of the results.

In future studies, validating the system on multicentre datasets and, if possible, testing it in prospective clinical scenarios to increase its generalisability will contribute to enhancing clinical applicability and reliability. Furthermore, testing the system in real-time clinical usage scenarios via a web-based interface presents an important area for expansion in terms of evaluating user interaction and decision-making process integration. Additionally, conducting analyses using longitudinal studies to predict disease progression and/or treatment response by tracking the same patients over time could increase the system’s clinical value. Finally, examining the relationship between segmentation and activation-based visualisations and clinical/laboratory indicators to validate the model outputs clinically could support the reliability of explainability.

## Figures and Tables

**Table 1:** Demographic and clinical characteristics of the study population.

Characteristics	Overall (*n* = 367)	axSpA (*n* = 193)	non-axSpA (*n* = 174)	*p*-Value
Age (years), mean ± SD	30.6 ± 7.2	32.0 ± 6.8	29.0 ± 7.5	<0.001 ^a^
Sex, *n* (%)				0.185 ^b^
Female	139 (37.9%)	67 (34.7%)	72 (41.4%)	
Male	228 (62.1%)	126 (65.3%)	102 (58.6%)	
Symptom duration (months), median (IQR)	20.0 (12–36)	23.0 (14–42)	18.0 (10–30)	0.015 ^c^
Symptom duration, *n* (%)				0.038 ^b^
Symptom duration < 5 years, *n* (%)	336 (91.6%)	171 (88.6%)	165 (94.8%)	
Symptom duration > 5 years, *n* (%)	31 (8.4%)	22 (11.4%)	9 (5.2%)	
HLA-B27 positive, *n* (%)	99 (27.0%)	87 (45.1%)	12 (6.9%)	<0.001 ^b^
CRP elevated, *n* (%)	209 (56.9%)	171 (88.6%)	38 (21.8%)	<0.001 ^b^
Inflammatory back pain, *n* (%)	203 (55.3%)	179 (92.7%)	24 (13.8%)	<0.001 ^b^
Chronic Back pain, *n* (%)	346 (94.3%)	180 (93.3%)	166 (95.4%)	0.382 ^b^
Arthritis, *n* (%)	113 (30.8%)	96 (49.7%)	17 (9.8%)	<0.001 ^b^
Enthesitis, *n* (%)	14 (3.8%)	12 (6.2%)	2 (1.1%)	0.012 ^b^
Uveitis, *n* (%)	16 (4.4%)	14 (7.3%)	2 (1.1%)	0.004 ^b^
Dactylitis, *n* (%)	16 (4.4%)	13 (6.7%)	3 (1.7%)	0.018 ^b^
Psoriasis, *n* (%)	4 (1.1%)	4 (2.1%)	0 (0.0%)	0.125 ^d^
Crohn’s/Ulcerative colitis, *n* (%)	2 (0.5%)	2 (1.0%)	0 (0.0%)	0.500 ^d^
Family history of SpA, *n* (%)	28 (7.6%)	24 (12.4%)	4 (2.3%)	<0.001 ^b^
Good response to NSAID, *n* (%)	96 (26.2%)	86 (44.6%)	10 (5.7%)	<0.001 ^b^

Notes: SD: Standard Deviation; IQR: Interquartile range; CRP: C-reactive protein; NSAID: Non-steroidal anti-inflammatory drugs. ^a^ Welch’s *t*-test was used for comparing mean ages. ^b^ Chi-square test was used for categorical variables. ^c^ Mann–Whitney U test was used for symptom duration (months). ^d^ Fisher’s exact test was used due to small cell frequencies (*n* < 5). *p* < 0.05 was considered statistically significant. Interquartile Range (25th–75th percentiles).

**Table 2:** Patient-level diagnostic performance results for the T1-WI sequence.

Model	AUC	95% CI (AUC)	Accuracy	Precision	Sensitivity	F1-Score	Specificity
**SacroNet**	0.900	0.865–0.935	0.851	0.833	0.897	0.864	0.800
**ResNet50**	0.822	0.778–0.866	0.783	0.810	0.769	0.789	0.800
**DenseNet121**	0.913	0.882–0.944	0.851	0.868	0.846	0.857	0.857
**VGG16**	0.920	0.891–0.949	0.878	0.941	0.820	0.876	0.942
**InceptionV3**	0.912	0.880–0.944	0.837	0.935	0.743	0.828	0.942

**Table 3:** Patient-level diagnostic performance results for the T2-WI sequence.

Model	AUC	95% CI (AUC)	Accuracy	Precision	Sensitivity	F1-Score	Specificity
**SacroNet**	0.717	0.662–0.772	0.650	1.000	0.382	0.553	1.000
**ResNet50**	0.575	0.520–0.630	0.616	0.720	0.529	0.610	0.730
**DenseNet121**	0.669	0.612–0.726	0.650	0.685	0.705	0.695	0.576
**VGG16**	0.608	0.552–0.664	0.600	0.692	0.529	0.600	0.692
**InceptionV3**	0.647	0.590–0.704	0.650	0.760	0.558	0.644	0.769

**Table 4:** Patient-level diagnostic performance results for the STIR sequence.

Model	AUC	95% CI (AUC)	Accuracy	Precision	Sensitivity	F1-Score	Specificity
**SacroNet**	0.733	0.688–0.778	0.728	0.720	0.815	0.765	0.625
**ResNet50**	0.686	0.635–0.737	0.671	0.826	0.500	0.622	0.875
**DenseNet121**	0.794	0.751–0.837	0.771	0.866	0.684	0.764	0.875
**VGG16**	0.782	0.739–0.825	0.742	0.833	0.657	0.735	0.843
**InceptionV3**	0.781	0.738–0.824	0.728	0.806	0.657	0.724	0.812

**Table 5:** Patient-level diagnostic performance results for the PD-WI sequence.

Model	95% CI (AUC)	AUC	Accuracy	Precision	Sensitivity	F1-Score	Specificity
**SacroNet**	0.634–0.736	0.685	0.696	0.722	0.787	0.753	0.565
**ResNet50**	0.558–0.668	0.613	0.625	0.800	0.484	0.603	0.826
**DenseNet121**	0.630–0.732	0.681	0.696	0.766	0.696	0.730	0.695
**VGG16**	0.721–0.809	0.765	0.750	0.731	0.909	0.810	0.521
**InceptionV3**	0.605–0.711	0.658	0.714	0.793	0.696	0.741	0.739

**Table 6:** Diagnostic performance results of the models that perform best at the patient level for all sequences.

Sequence	Model	AUC	95% CI (AUC)	Accuracy	Precision	Sensitivity	F1-Score	Specificity
**T1-WI**	VGG16	0.920	0.891–0.949	0.878	0.941	0.820	0.876	0.942
**T2-WI**	DenseNet121	0.669	0.612–0.726	0.650	0.685	0.705	0.695	0.576
**STIR**	DenseNet121	0.794	0.751–0.837	0.771	0.866	0.684	0.764	0.875
**PD-WI**	VGG16	0.765	0.721–0.809	0.750	0.731	0.909	0.810	0.521

**Table 7:** Patient-level diagnostic performance results for multimodal.

Model	AUC	95% CI (AUC)	Accuracy	Precision	Sensitivity	F1-Score	Specificity
**Multimodal**	0.905	0.871–0.939	0.837	0.864	0.820	0.842	0.850

**Table 8:** Clinical variables ranked by their ORs in the logistic regression-based risk model.

Rank	Clinical Variable	Odds Ratio (OR)
1	Inflammatory back pain (IBP)	7.612
2	Elevated CRP	5.361
3	HLA-B27	2.655
4	Arthritis	1.984
5	Family history of SpA	1.858
6	Uveitis	1.775
7	Psoriasis	1.539
8	Dactylitis	1.251
9	Crohn’s disease/ulcerative colitis	1.152
10	Enthesitis	0.915

**Table 9:** Examples related to the fusion approach.

Patient ID	$P_{image}$ (VGG16- T1-WI)	$P_{clinical}$	$P_{final}$	Optimal Decision Threshold	Class
Active_56	0.67 (non-axSpA)	0.83	(0.67 × 0.6) + (0.83 × 0.4) = 0.73	0.73 > 0.70	axSpA
Active_81	0.65 (non-axSpA)	0.80	(0.65 × 0.6) + (0.80 × 0.4) = 0.71	0.71 > 0.70	axSpA
Active_171	0.83	0.87	(0.83 × 0.6) + (0.87 × 0.4) = 0.84	0.84 > 0.70	axSpA
non_axSpA_141	0.26	0.09	(0.26 × 0.6) + (0.09 × 0.4) = 0.19	0.19 < 0.70	non-axSpA

**Table 10:** Segmentation performance of models for the T2-WI sequence (mean ± standard deviation).

Model	DSC	95% CI (DSC)	IoU	Precision	Sensitivity	HD95 (px)
**U-Net**	0.660 ± 0.032	0.631–0.686	0.625 ± 0.035	0.684 ± 0.030	0.828 ± 0.044	28.88 ± 2.78
**ResUNet**	0.508 ± 0.010	0.479–0.536	0.473 ± 0.011	0.513 ± 0.006	0.842 ± 0.037	45.54 ± 2.01
**AttUNet**	0.653 ± 0.058	0.624–0.681	0.617 ± 0.058	0.672 ± 0.036	0.845 ± 0.051	31.38 ± 5.94
**UNet++**	0.652 ± 0.068	0.628–0.680	0.616 ± 0.068	0.656 ± 0.058	0.852 ± 0.046	30.49 ± 5.70
**TransUNet**	0.651 ± 0.056	0.625–0.678	0.615 ± 0.058	0.653 ± 0.057	0.849 ± 0.044	29.35 ± 5.92
**MDC-UNet**	0.682 ± 0.038	0.655–0.708	0.646 ± 0.037	0.713 ± 0.032	0.823 ± 0.042	26.21 ± 3.31

**Table 11:** Segmentation performance of models for the STIR sequence (mean ± standard deviation).

Model	DSC	95% CI (DSC)	IoU	Precision	Sensitivity	HD95 (px)
**U-Net**	0.725 ± 0.042	0.699–0.749	0.690 ± 0.046	0.731 ± 0.042	0.888 ± 0.016	22.48 ± 4.55
**ResUNet**	0.626 ± 0.109	0.597–0.654	0.593 ± 0.107	0.633 ± 0.112	0.880 ± 0.007	33.86 ± 12.39
**AttUNet**	0.725 ± 0.032	0.701–0.751	0.691 ± 0.036	0.741 ± 0.035	0.877 ± 0.021	22.77 ± 3.24
**UNet++**	0.734 ± 0.029	0.710–0.759	0.700 ± 0.029	0.748 ± 0.019	0.885 ± 0.029	22.25 ± 2.60
**TransUNet**	0.737 ± 0.060	0.712–0.761	0.701 ± 0.060	0.744 ± 0.058	0.886 ± 0.023	19.44 ± 6.61
**MDC-UNet**	0.751 ± 0.021	0.726–0.776	0.716 ± 0.019	0.753 ± 0.005	0.885 ± 0.025	19.25 ± 0.86

## Data Availability

The data presented in this study are available on request from the corresponding author due to privacy and ethical restrictions concerning patient confidentiality and the sensitive nature of medical imaging data.

## Supplementary Materials

The following supporting information can be downloaded at: https://www.mdpi.com/article/10.3390/diagnostics16071037/s1, Section S1: Inclusion/exclusion criteria and group definitions, Section S2: Data Preprocessing and Training Protocol, Table S1: Classification-based studies, Table S2: Studies combining classification and segmentation, Supplementary Table S3: Confusion matrix, Supplementary Table S4: Formulas for Calculating Performance Metrics for Classification, Supplementary Table S5: Formulas for Calculating Performance Metrics for Segmentation, Figure S1: DenseNet121 T2-WI Confusion Matrix, Figure S2: DenseNet121 T2-WI ROC Curve, Figure S3: DenseNet121-STIR Confusion Matrix, Figure S4: DenseNet121-STIR ROC Curve, Figure S5: VGG16 PD-WI Confusion Matrix, Figure S6: VGG16 PD-WI ROC Curve. Reference list from Supplementary Materials is cited here as Refs. [12,26,27,31,32,38,39,40,41,42,43,44,45,46,47,48,49,50,51,52,53,54,55].

## Abbreviations

The following abbreviations are used in this manuscript:

axSpA	Axial Spondyloarthritis
AS	Ankylosing Spondylitis
ASAS	Assessment of SpondyloArthritis International Society
AUC	Area Under the Curve
BME	Bone Marrow oedema
CBP	Chronic Back Pain
CDSS	Clinical Decision Support System
CI	Confidence Interval
CNN	Convolutional Neural Network
CRP	C-Reactive Protein
DICOM	Digital Imaging and Communications in Medicine
DL	Deep Learning
DSC	Dice Similarity Coefficient
Grad-CAM	Gradient-Weighted Class Activation Mapping
GT	Ground Truth
HD95	95th Percentile Hausdorff Distance
HLA-B27	Human Leukocyte Antigen B27
IBP	Inflammatory Back Pain
LR	Logistic Regression
MDC-UNet	Multi-scale Dilated Contextual U-Net
ML	Machine Learning
MRI	Magnetic Resonance Imaging
NSAIDs	Non-steroidal Anti-Inflammatory Drugs
OMERACT	Outcome Measures in Rheumatology
OR	Odds Ratio
PD-WI	Proton Density-Weighted Imaging
ResUNet	Residual U-Net
ROC	Receiver Operating Characteristic
ROI	Region of Interest
SacroNet	Proposed classification model
SIJ	Sacroiliac Joint
SpA	Spondyloarthritis
STIR	Short Tau Inversion Recovery
T1-WI	T1-weighted imaging
T2-WI	T2-weighted imaging
TransUNet	Transformer-based U-Net
