# Supplementary material for: A Comprehensive Clinical Decision Support System for the Early Diagnosis of Axial Spondyloarthritis: Multi-Sequence MRI, Clinical Risk Integration, and Explainable Segmentation
Source: Diagnostics (Basel). 2026 Mar 30;16(7):1037. doi: 10.3390/diagnostics16071037 (PMC13072811; doi:10.3390/diagnostics16071037)
Supplement: Supplementary file 1 [file diagnostics-16-01037-s001.zip › diagnostics-4186521-supplementary.pdf]

A Comprehensive Clinical Decision Support System for the Early Diagnosis of Axial Spondyloarthritis: Multi-Sequence MRI, Clinical Risk Integration, and Explainable Segmentation

ELECTRONIC SUPPLEMENTARY MATERIAL

<u>Supplementary Sections</u>

Supplementary Section S1: Inclusion and exclusion criteria and group definitions

The criteria for inclusion of participants in the study were determined as follows:

(A) A history of chronic low back pain lasting longer than three months,

(B) Being over 18 years of age and having a symptom onset age below 45 years,

(C) Having undergone SIJ MRI with T1-WI, T2-WI, STIR, and PD-WI sequences between January 2020 and December 2022.

Exclusion criteria were defined as follows:

(A) Traumatic or insufficiency-related pelvic fractures

(B) Previously administered radiotherapy

(C) Tumoural lesions extending to the SIJs

No pre-screening based on image quality was performed in the study.

Group definitions:

Participants meeting the inclusion/exclusion criteria were divided into two groups based on clinical records and imaging assessments:

axSpA group: Based on SIJ MRG findings and clinical evaluation, cases classified in favour of axSpA according to ASAS classification criteria formed this group.

CBP control group (non-axSpA): Participants who had experienced back pain for at least three months, consulted the rheumatology department, and underwent SIJ MRI for this reason; however, those who were not evaluated in favour of axSpA based on clinical and/or imaging findings and did not meet the ASAS classification criteria formed the control group. This group represents the symptomatic presentation population in which axSpA differential diagnosis is made in clinical practice and has been classified as "non-axSpA" without excluding other mechanical/degenerative causes that may accompany back pain.

Supplementary Section S2: Data Preprocessing and Training Protocol

Detailed technical information regarding the data preprocessing and model training processes applied to the MRI data is presented in this section. MRIs obtained in DICOM format were converted to two-dimensional PNG slices as part of data standardisation; the original slices and binary masks were rescaled to 256×256 dimensions. The dataset was separated on a patient basis to prevent data

leakage. Accordingly, all slices belonging to each patient were ensured to be included in only one dataset. For intensity normalisation, a percentile clipping process of 1–99% was applied to each slice, and the data were pulled into the [0,1] range and converted to an 8-bit (0–255) space.

To enhance the generalisation ability of the models, comprehensive data augmentation strategies (horizontal flipping, rotation, elastic deformation, noise addition, etc.) were applied using the Albumentations library [1].

The classification models were developed using the TensorFlow/Keras library. All models were trained for 100 epochs under the same training protocol and hyperparameter settings, with a batch size of 16; the top layers of ImageNet pre-trained networks were fine-tuned in a controlled manner to adapt to MRI data. The Adam algorithm was used in the optimisation process. The Binary Focal Cross-Entropy loss function was preferred to reduce class imbalance. Model performance was monitored using validation data; early stopping and learning rate reduction strategies were applied based on the validation AUC metric.

Segmentation models were developed using the PyTorch library. The training process was carried out for 50 epochs with a batch size of 64. AdamW was chosen as the optimisation algorithm, and the OneCycleLR scheduler was used. To minimise the class imbalance problem, the improved dice + BCE combination was used as the loss function. The BCE component was enhanced with positive class weight (pos_weight=10) and label smoothing=0.1. Each experiment was repeated with three random seed values, and the results were reported as the mean ± standard deviation.

All experimental processes were performed on GPU-supported workstations, with classification models trained on an NVIDIA GeForce GTX 1660 Ti GPU and segmentation models trained on an NVIDIA Quadro RTX 8000 GPU.

Supplementary Tables

Supplementary Table S1: Classification-based studies

Table S1 presents a comparative overview of classification-based studies for axSpA diagnosis, considering the type of data used, imaging modality, MRI sequence, SIJ ROI/Localization approach, and artificial intelligence methods employed.

Supplementary Table S1. Classification-based studies

Year	Authors	Modality	Sequence	SIJ ROI / Localization	Method
**2020**	A. Deodhar et al. [2]	Clinical and administrative data	-	-	ML
**2020**	Y. B. Joo et al. [3]	Clinical and laboratory data, radiographic scores	-	-	ML
**2020**	R. Castro-Zunti et al. [4]	CT	-	-	ML+DL
**2020**	M. C. Faleiros et al. [5]	MRI	STIR	Manuel ROI	ML
**2021**	K. K. Bressem et al. [6]	X-ray	X-ray	-	CNN
**2022**	K. K. Bressem et al. [7]	MRI	T1, STIR	-	CNN
**2023**	M. Triantafyllou et al. [8]	MRI	STIR+PD	Manuel ROI	Radiomics + ML
**2023**	M. Zheng et al. [9]	MRI	T1	Manuel ROI	Radiomics + ML
**2023**	N. P. Tas et al. [10]	MRI	STIR	-	CNN+kNN
**2023**	S. Lee et al. [11]	MRI	STIR	ROI Bounding	CNN (VGG-19)

				box	
**2023**	S. Turk et al. [12]	MRI	T1, STIR	Manuel ROI	JointNet (ensemble CNN)
**2024**	S. H. Ahammad et al. [13]	Clinical + demographic data	-	-	ML
**2024**	E. Canayaz et al. [14]	MRI	STIR	Manuel ROI	ML
**2025**	S. Uzelaltinbulat et al. [15]	MRI	T1, T2, STIR, T2-FS	-	CNN
**2025**	Z. Xie et al. [16]	MRI + Clinical	T1, T2, FS	ROI Bounding box	DL + ML fusion

Supplementary Table S2: Studies combining classification and segmentation

Table S2 presents a comparative overview of studies that address both classification and segmentation processes. Table S2 reveals that the literature largely focuses on U-Net and derivative architectures for these approaches and that STIR and T1-WI MRI sequences are commonly used for inflammation assessment.

Supplementary Table S2. Studies combining classification and segmentation

**Year**	**Authors**	**Modality**	**Sequence**	**SIJ ROI / Localization**	**Method**
**2021**	Q. Han et al. [17]	MRI	STIR	Automated	3D U-Net + CNN
**2022**	K. Y. Y. Lin et al.[18]	MRI	STIR	Automated	Attention U-Net
**2023**	K. Zhang et al. [19]	CT	CT	Automated	nnU-Net + CNN
**2023**	A. Bordner et al. [20]	MRI	T1, STIR	Automated	Mask-RCNN
**2024**	Y. Lin et al. [21]	MRI	STIR	Automated	Attention U-Net
**2024**	K. Zhang et al. [22]	MRI	T1, STIR	Automated	3D U-Net + CNN
**2024**	J. Nicolaes et al. [23]	MRI	T1, STIR	Automated	U-Net+CNN

Supplementary Table S3: Confusion matrix

The confusion matrix, shown in Supplementary Table S3, is a basic analytical tool used to evaluate and compare the classification performances of DL models. The confusion matrix summarizes the relationship between the model's predictions and the true labels and consists of four basic components: True Positive (TP), False Negative (FN), True Negative (TN), False Positive (FP).

Supplementary Table S3. Confusion matrix

		**Prediction Class**	
		**Positive**	**Negative**
**Actual Class**	**Positive**	*TP True Positive (TP):* The number of instances that the model predicted as positive and were actually positive.	*False Negative (FN):* The number of instances that the model predicted as negative but were actually positive.
	**Negative**	*False Positive (FP):* The number of instances that the model predicted as positive but were actually negative.	*True Negative (TN):* The number of instances that the model predicted as negative and were actually negative.

Supplementary Table S4: Formulas for Calculating Performance Metrics for Classification

Classification Metrics

The four components provided in Supplementary Table S3 play a critical role in calculating fundamental performance metrics such as Accuracy, Sensitivity, Specificity, Precision, and F1 Score to evaluate the model's performance. The calculation formulas for these metrics are provided in Supplementary Table S4.

Supplementary Table S4. Formulas for Calculating Performance Metrics for Classification

Accuracy	=	(TP+TN) / (TP+TN+FP+FN)
Sensitivity	=	TP / (TP + FN)
Specificity	=	TN / (TN + FP)
Precision	=	TP / (TP + FP)
F1 Scores	=	2 × (Precision × Sensitivity) / (Precision + Sensitivity)
AUC	=	AUC = $\int_0^1 TPR\ (FPR)\ dFPR$<br>TPR (True Positive Rate): Y-axis (Sensitivity).<br>FPR (False Positive Rate): X-axis (1-Specificity).

Supplementary Table S5: Formulas for Calculating Performance Metrics for Segmentation

Segmentation Metrics

For the table in Supplementary Table S5, let G represent the ground truth mask and P represent the prediction mask.

Supplementary Table S5. Formulas for Calculating Performance Metrics for Segmentation

Dice Similarity Coefficient (DSC)	Measures the spatial overlap between the prediction and the ground truth.	DSC (G, P) = (2 * ∣G ∩ P∣) / (∣G∣ + ∣P∣)
Intersection over Union (IoU)	Also known as the Jaccard Index, it measures the ratio of intersection to union.	IoU (G, P) = ∣G ∩ P∣ / ∣G ∪ P∣
Precision	The proportion of predicted pixels that are actually part of the ground truth.	Precision = ∣G ∩ P∣ / ∣P∣
Sensitivity	The proportion of ground truth pixels that are correctly predicted by the model.	Sensitivity = ∣G ∩ P∣ / ∣G∣
95% Hausdorff Distance (HD95)	Measures the maximum distance between the boundaries of G and P. The 95th percentile is used to mitigate the impact of outliers and noise in segmentation boundaries.	

<u>**Supplementary Figures**</u>

Supplementary Figure S1: DenseNet121 T2-WI Confusion Matrix

Supplementary Figure S2: DenseNet121 T2-WI ROC Curve

The confusion matrix for the DenseNet121 model, which demonstrated the best performance in the T2-WI MRI sequence, is presented in Figure S1, while the ROC curve is presented in Figure S2.

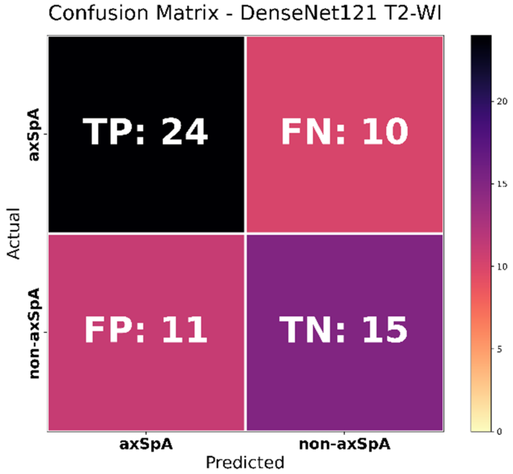

Figure S1. DenseNet121 T2-WI Confusion Matrix

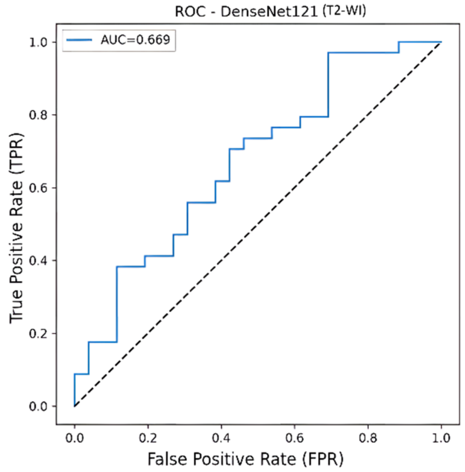

Figure S2. DenseNet121 T2-WI ROC Curve

Supplementary Figures S3: DenseNet121-STIR Confusion Matrix

Supplementary Figures S4: DenseNet121-STIR ROC Curve

The confusion matrix for the DenseNet121 model, which showed the best performance in the STIR MRI sequence, is presented in Figure S3, and the ROC curve is presented in Figure S4.

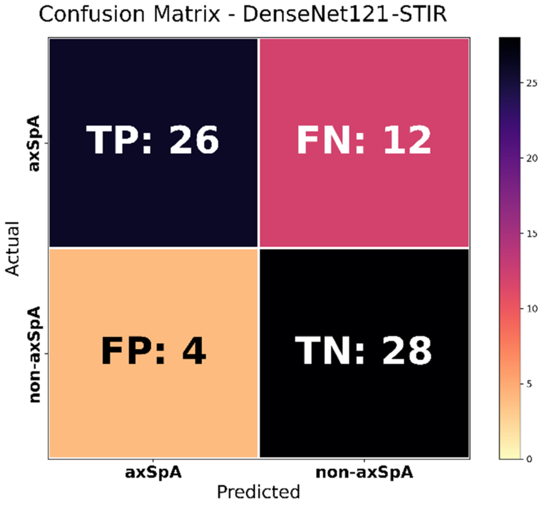

Figure S3: DenseNet121-STIR Confusion Matrix

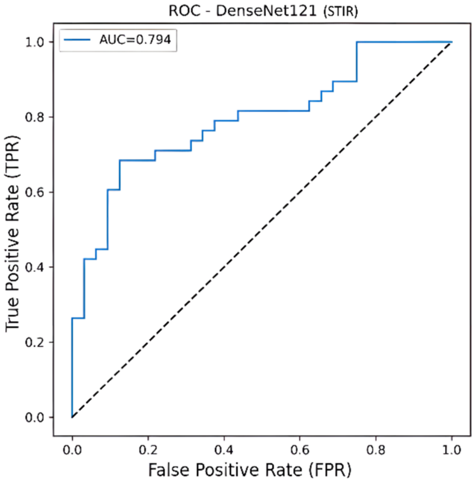

Figure S4: DenseNet121-STIR ROC Curve

Supplementary Figures S5: VGG16 PD-WI Confusion Matrix

Supplementary Figures S6: VGG16 PD-WI ROC Curve

The confusion matrix for the VGG16 model, which demonstrated the best performance in the PD MRI sequence, is presented in Figure S5, while the ROC curve is presented in Figure S6.

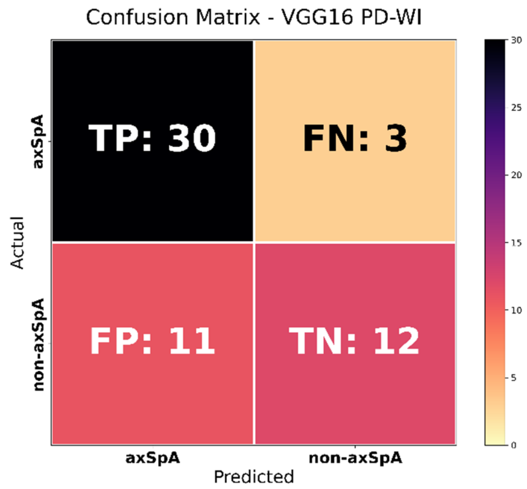

Figure S5: VGG16 PD-WI Confusion Matrix

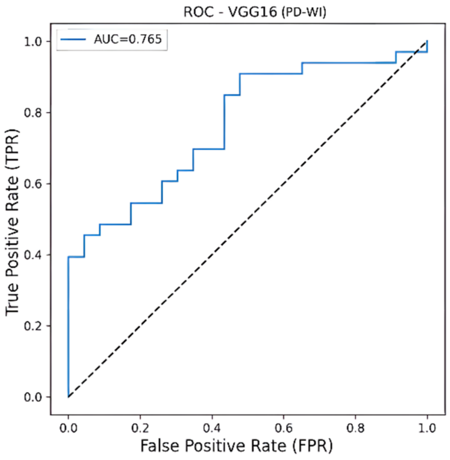

Figure S6: VGG16 PD-WI ROC Curve

References

1. Buslaev, A.; Iglovikov, V.I.; Khvedchenya, E.; Parinov, A.; Druzhinin, M.; Kalinin, A.A. Albumentations: Fast and Flexible Image Augmentations. *Information* **2020**, *11*, doi:https://doi.org/10.3390/info11020125.
2. Deodhar, A.; Rozycki, M.; Garges, C.; Shukla, O.; Arndt, T.; Grabowsky, T.; Park, Y. Use of machine learning techniques in the development and refinement of a predictive model for early diagnosis of ankylosing spondylitis. *Clin Rheumatol* **2020**, *39*, 975-982, doi:https://doi.org/10.1007/s10067-019-04553-x.
3. Joo, Y.B.; Baek, I.W.; Park, Y.J.; Park, K.S.; Kim, K.J. Machine learning-based prediction of radiographic progression in patients with axial spondyloarthritis. *Clin Rheumatol* **2020**, *39*, 983-991, doi:https://doi.org/10.1007/s10067-019-04803-y.
4. Castro-Zunti, R.; Park, E.H.; Choi, Y.; Jin, G.Y.; Ko, S.-b. Early detection of ankylosing spondylitis using texture features and statistical machine learning, and deep learning, with some patient age analysis. *Computerized Medical Imaging and Graphics* **2020**, *82*, 101718, doi:https://doi.org/10.1016/j.compmedimag.2020.101718.
5. Faleiros, M.C.; Nogueira-Barbosa, M.H.; Dalto, V.F.; Júnior, J.R.F.; Tenório, A.P.M.; Luppino-Assad, R.; Louzada-Junior, P.; Rangayyan, R.M.; de Azevedo-Marques, P.M. Machine learning techniques for computer-aided classification of active inflammatory sacroiliitis in magnetic resonance imaging. *Advances in Rheumatology* **2020**, *60*, 25, doi:https://doi.org/10.1186/s42358-020-00126-8.
6. Bressem, K.K.; Vahldiek, J.L.; Adams, L.; Niehues, S.M.; Haibel, H.; Rodriguez, V.R.; Torgutalp, M.; Protopopov, M.; Proft, F.; Rademacher, J.; et al. Deep learning for detection of radiographic sacroiliitis: achieving expert-level performance. *Arthritis Research & Therapy* **2021**, *23*, 106, doi:https://doi.org/10.1186/s13075-021-02484-0.
7. Bressem, K.K.; Adams, L.C.; Proft, F.; Hermann, K.G.A.; Diekhoff, T.; Spiller, L.; Niehues, S.M.; Makowski, M.R.; Hamm, B.; Protopopov, M.; et al. Deep Learning Detects Changes Indicative of Axial Spondyloarthritis at MRI of Sacroiliac Joints. *Radiology* **2022**, *305*, 655-665, doi:https://doi.org/10.1148/radiol.212526.
8. Triantafyllou, M.; Klontzas, M.E.; Koltsakis, E.; Papakosta, V.; Spanakis, K.; Karantanas, A.H. Radiomics for the Detection of Active Sacroiliitis Using MR Imaging. *Diagnostics (Basel)* **2023**, *13*, doi:https://doi.org/10.3390/diagnostics13152587.

9. Zheng, M.; Zhu, G.; Chen, D.; Xiao, Q.; Lei, T.; Ye, C.; Pan, C.; Miao, S.; Ye, L. T1-weighted images-based radiomics for structural lesions evaluation in patients with suspected axial spondyloarthritis. *Radiol Med* **2023**, *128*, 1398-1406, doi:https://doi.org/10.1007/s11547-023-01717-3.
10. Tas, N.P.; Kaya, O.; Macin, G.; Tasci, B.; Dogan, S.; Tuncer, T. ASNET: A Novel AI Framework for Accurate Ankylosing Spondylitis Diagnosis from MRI. *Biomedicines* **2023**, *11*, 2441, doi:https://doi.org/10.3390/biomedicines11092441.
11. Lee, S.; Jeon, U.; Lee, J.H.; Kang, S.; Kim, H.; Lee, J.; Chung, M.J.; Cha, H.S. Artificial intelligence for the detection of sacroiliitis on magnetic resonance imaging in patients with axial spondyloarthritis. *Front Immunol* **2023**, *14*, 1278247, doi:https://doi.org/10.3389/fimmu.2023.1278247.
12. Turk, S.; Demirkaya, A.; Turali, M.Y.; Hepdurgun, C.; Dar, S.U.; Karabulut, A.K.; Azizova, A.; Orman, M.; Tamsel, I.; Aydingoz, U. Jointnet: A deep model for predicting active sacroiliitis from sacroiliac joint radiography. *arXiv preprint arXiv:2301.10769* **2023**, doi:http://dx.doi.org/10.48550/arXiv.2301.10769.
13. Ahammad, S.H.; Jayaraj, R.; Shibu, S.; Sujatha, V.; Prathima, C.; Leo, L.M.; Prabu, R.T.; Hossain, M.A.; Rashed, A.N.Z. Advanced model based machine learning technique for early stage prediction of ankylosing spondylitis under timely analysis with featured textures. *Multimedia Tools and Applications* **2024**, doi:https://doi.org/10.1007/s11042-024-18236-6.
14. Canayaz, E.; Altikardes, Z.A.; Unsal, A.; Korkmaz, H.; Gok, M. Development and validation of machine learning algorithms for early detection of ankylosing spondylitis using magnetic resonance images. *Technol Health Care* **2025**, *33*, 1182-1198, doi:https://doi.org/10.1177/09287329241297887.
15. Uzelaltinbulat, S.; Kucukciloglu, Y.; Ilhan, A.; Mirzaei, O.; Sekeroglu, B. Diagnosis of sacroiliitis using MR images with a simplified custom deep learning model. *The Journal of Supercomputing* **2025**, *81*, 781, doi:https://doi.org/10.1007/s11227-025-07280-0.
16. Xie, Z.; Chen, Z.; Yang, Q.; Ye, Q.; Li, X.; Xie, Q.; Liu, C.; Lin, B.; Han, X.; He, Y.; et al. Enhanced diagnosis of axial spondyloarthritis using machine learning with sacroiliac joint MRI: a multicenter study. *Insights into Imaging* **2025**, *16*, 91, doi:https://doi.org/10.1186/s13244-025-01967-x.
17. Han, Q.; Lu, Y.; Han, J.; Luo, A.; Huang, L.; Ding, J.; Zhang, K.; Zheng, Z.; Jia, J.; Liang, Q.; et al. Automatic quantification and grading of hip bone marrow oedema in ankylosing spondylitis based on deep learning. *Modern Rheumatology* **2021**, *32*, 968-973, doi:https://doi.org/10.1093/mr/roab073.
18. Lin, K.Y.Y.; Peng, C.; Lee, K.H.; Chan, S.C.W.; Chung, Ho Y. Deep learning algorithms for magnetic resonance imaging of inflammatory sacroiliitis in axial spondyloarthritis. *Rheumatology* **2022**, *61*, 4198-4206, doi:https://doi.org/10.1093/rheumatology/keac059.
19. Zhang, K.; Luo, G.; Li, W.; Zhu, Y.; Pan, J.; Li, X.; Liu, C.; Liang, J.; Zhan, Y.; Zheng, J.; et al. Automatic Image Segmentation and Grading Diagnosis of Sacroiliitis Associated with AS Using a Deep Convolutional Neural Network on CT Images. *Journal of Digital Imaging* **2023**, *36*, 2025-2034, doi:https://doi.org/10.1007/s10278-023-00858-1.
20. Bordner, A.; Aouad, T.; Medina, C.L.; Yang, S.; Molto, A.; Talbot, H.; Dougados, M.; Feydy, A. A deep learning model for the diagnosis of sacroiliitis according to Assessment of SpondyloArthritis International Society classification criteria with magnetic resonance imaging. *Diagn Interv Imaging* **2023**, *104*, 373-383, doi:https://doi.org/10.1016/j.diii.2023.03.008.
21. Lin, Y.; Chan, S.C.W.; Chung, H.Y.; Lee, K.H.; Cao, P. A deep neural network for MRI spinal inflammation in axial spondyloarthritis. *Eur Spine J* **2024**, doi:https://doi.org/10.1007/s00586-023-08099-0.
22. Zhang, K.; Liu, C.; Pan, J.; Zhu, Y.; Li, X.; Zheng, J.; Zhan, Y.; Li, W.; Li, S.; Luo, G.; et al. Use of MRI-based deep learning radiomics to diagnose sacroiliitis related to axial spondyloarthritis. *Eur J Radiol* **2024**, *172*, 111347, doi:https://doi.org/10.1016/j.ejrad.2024.111347.
23. Nicolaes, J.; Tselenti, E.; Aouad, T.; López-Medina, C.; Feydy, A.; Talbot, H.; Hoepken, B.; de Peyrecave, N.; Dougados, M. Performance analysis of a deep-learning algorithm to detect the presence of inflammation in MRI of sacroiliac joints in patients with axial spondyloarthritis. *Ann Rheum Dis* **2025**, *84*, 60-67, doi:https://doi.org/10.1136/ard-2024-225862.
